# Mechanobiology in Cells and Tissues

**DOI:** 10.3390/ijms24108564

**Published:** 2023-05-10

**Authors:** Sabata Martino

**Affiliations:** Department of Chemistry, Biology and Biotechnologies, University of Perugia, Via del Giochetto, 06122 Perugia, Italy; sabata.martino@unipg.it; Tel.: +39-0755-857442

This Editorial is a comment on the success of the Special Issue “Mechanobiology in Cells and Tissues” published in the *International Journal of Molecular Sciences* [1].

The field of mechanobiology began in 1990 with the application of the concept of cell “tensegrity” (tensional integrity) to the biomolecular dynamic cross-talk by which living cells respond to the external physical stimuli orchestrated by the extracellular matrix (ECM) and remodel their cytoskeleton, and by this activate a signal cascade collected by the nucleus, where a tailored gene expression program is activated to control the cell “decision making” and functions [2,3]. Indeed, mechanobiology is as old as living organisms. In the human body, biophysical stimuli exerted by ECM or fluids surrounding the cells are necessary for tissue development and maintenance of tissue homeostasis, morphology, and function throughout life [4,5,6]. Therefore, understanding how physical forces are applied to cells and identifying the types of proteins involved in the phenomenon is currently challenging. Now we know that independently of the type of mechanical forces (e.g., compression, stiffness, elasticity, membrane tension, hydrostatic pressure, and shear stress), physical cues propagation is the fastest signaling route, and these are rapidly translated into biochemical pathways and metabolic responses [5]. The proteins involved in collecting and transducing the physical stimuli are called ‘mechanosensors’ and include transmembrane proteins (e.g., integrins and ion channels) and intracellular proteins (e.g., focal adhesion proteins, cytoskeleton, nucleoskeleton, and soluble proteins specifically responsive to physical signals) organized in molecular complexes which are players of the “mechanosensing” and “mechanotransduction” pathways, respectively. Hence, when physical forces are applied to cell membranes, the proteins of mechanosensing pathways sense the mechanical stimuli and transmit them to the proteins of the mechanotransduction pathways.

The significance of mechanosensing and mechanotransduction processes in the cell function is highlighted by growing evidence correlating the alterations of such biochemical pathways with disease development and progression, as well as with the advancing fields of regenerative and personalized medicine [7]. Thus, the mechanical properties of the bone matrix have a critical role in the maintenance of healthy bone tissue and regeneration, even in the case of osteosarcoma, where current therapies directly target mechanosensor elements of the tumor or combine the latter with chemotherapy agents improving the chemosensitivity [8], or recreate the bone matrix by using innovative biomaterials which mimic the bone microenvironment. Physical stimuli (e.g., tension and pressure) are also recognized as regulators of mechanosensor proteins central for the development and spatiotemporal organization of the nervous system [9] and brain diseases (e.g., traumatic brain injury, neurodegenerative diseases, and neuroblastoma [10]) when those pathways are aberrant. Mechanobiology pathways are also essential for cardiac cells’ function over their lifespan [11,12], and therefore, innovative therapeutic approaches attempt to regenerate the damaged tissue by re-establishing the mechanical microenvironment (e.g., stiffness range, topography, and viscoelasticity). Furthermore, dysfunction of cell mechanobiology machinery is now associated with cancer development and expansion, and therefore, mechanosensor proteins are explored as cancer diagnostic markers in cell body fluid samples, as well as anti-tumor targets [13,14].

The papers in the Special Issue “Mechanobiology in Cells and Tissues” explored the multidisciplinary aspects of mechanobiology and highlighted the role of cell mechanobiology in physiology and pathology.

Some authors elucidated specific mechanotransduction signaling.

Reiprich and co-authors demonstrated the activation of the hyaluronan synthases in bone marrow human mesenchymal stem exposed to a fluid shear stress of 10 Pa, providing a relevant contribution to understanding the role of hyaluronic acid in the bone matrix and its application in bone regeneration [15].

A.D. Bakker et al. showed that the exposure of murine Calvaria 3T3-E1 osteoblast cell lines to 1 h of pulsating fluid flow (PFF) caused fast cytoskeletal reorganization and changes in the cell and nucleus morphology and volume, together with the increase in the expression of genes and proteins involved in the mechanotransduction process [16]. The authors further provided insight into the mechanisms, demonstrating that the appropriated modulation of PFF stimulation to MC3T3-E1 pre-osteoblasts cell lines with the release of bioactive molecules stimulated the expression of osteogenic genes [17,18].

In the same topic, S. Carelli et al. explored the effect of the mechanical stimuli exerted by the Nichoid 3D scaffold on neural stem cells [19]. The miniaturized architecture of the scaffold recapitulated the niche of neural stem cells. However, the mechanical stimuli of the structure caused the alteration of specific cellular signaling correlating with mechanotransduction pathways from the plasma membrane to the cytoskeleton, cell metabolism, and gene expression. Thus, the authors warned about the careful regulation of mechanical stimuli to avoid aberrant effects on the stem cells’ metabolic and genetic response [19].

Other authors focused on the development of mechanobiology tools for tissue engineering applications.

The work by Clausen-Schaumann and co-authors presented an innovative optoelectronic micro-indenter consisting of Fiber Bragg Grating (FBG) containing a 25 mm long FBG within a photosensitive optical fiber [20]. The equipment detected tiny changes in articular cartilage stiffness, resulting in a sensitive tool for the diagnosis of cartilage degeneration caused by early-stage osteoarthritis [20].

Sang Eon Par and co-authors developed more proper procedures for the culture and expansion of mesenchymal stem cells to maintain stemness properties and therapeutic efficacy using mechanical stimuli. They demonstrated an increase in cell proliferation in Wharton’s jelly-derived mesenchymal stem cells exposed to pressure stimuli during the primary step of culture without causing alteration changes in the stemness biology [21].

Other authors give a comprehensive overview of cell and tissue mechanobiology aspects.

The work of Cobo and co-authors discussed the mechanotransduction mechanisms by which sensory corpuscles, localized in the skin of vertebrates, sense various mechanical stimuli (e.g., light brush, touch, pressure, stretch, and vibration) and transmit them to mechanosensory neurons, through Aβ, Aδ, and C nerve fibers. In particular, the work examined the role of mechanosensing ion channels localized in the axon and the periaxonal cells of sensory corpuscles, including the activity of Piezo channels and the superfamily of the transient receptor potential channels [22].

The review of Kar Wey Yong et al. documented the advancements of mechanobiology in bone tissue engineering approaches. The work discussed the relevance of mechanical stimuli in stem cell implantation for bone repair, highlighting the advantage of mechanical loading factors (compression, perfusion, vibration, and stretching) in the therapeutic efficacy for the human mesenchymal stem cells implanted in damaged bone, together with the potential mechanotransduction pathways activated in the process [23].

Antonios N. Gargalionis and co-authors summarized twenty years of findings on polycystin-1 and polycystin-2, two proteins responsive to changes in the extracellular mechanical cues, and whose dysfunctions are well associated with the formation of cysts in kidney cells [24]. The authors also point to the involvement of polycystins in the pathogenic mechanotransduction in other diseases, such as cancer, cardiovascular deficiencies, bone degeneration, and inflammations. In this regard, the same group has recently shown the implication of polycystin-1 in glioblastoma mechanobiology [25].

Our group also provided an overview of the molecular basis of mechanosensing and mechanotransduction pathways, and of their role in the cross-talk between stem cells and microenvironments, and how these dynamic interplays are critical in the control of stem cell identity and function, including their therapeutic application in regenerative medicine [2]. To this end, the relevance of the ex vivo mechanobiology model and the role of computational tools have been well emphasized and discussed.

In conclusion, the articles in the Special Issue on “Mechanobiology in Cells and Tissues” covered a collection of methods, concepts, and molecular findings, and the high number of researchers that have viewed and downloaded the published papers emphasizes the contribution to the knowledge on mechanobiology [1].
